# Perception of stochastically undersampled sound waveforms: a model of auditory deafferentation

**DOI:** 10.3389/fnins.2013.00124

**Published:** 2013-07-16

**Authors:** Enrique A. Lopez-Poveda, Pablo Barrios

**Affiliations:** ^1^Unidad de Audición Computacional y Psicoacústica, Instituto de Neurociencias de Castilla y León, Universidad de SalamancaSalamanca, Spain; ^2^Grupo de Audiología, Instituto de Investigación Biomédica de SalamancaSalamanca, Spain; ^3^Departamento de Cirugía, Facultad de Medicina, Universidad de SalamancaSalamanca, Spain; ^4^Unidad de Foniatría, Logopedia y Audiología, Hospital Universitario de SalamancaSalamanca, Spain

**Keywords:** auditory deafferentation, auditory aging, hearing loss, stochastic sampling, model, vocoder, information theory

## Abstract

Auditory deafferentation, or permanent loss of auditory nerve afferent terminals, occurs after noise overexposure and aging and may accompany many forms of hearing loss. It could cause significant auditory impairment but is undetected by regular clinical tests and so its effects on perception are poorly understood. Here, we hypothesize and test a neural mechanism by which deafferentation could deteriorate perception. The basic idea is that the spike train produced by each auditory afferent resembles a stochastically digitized version of the sound waveform and that the quality of the waveform representation in the whole nerve depends on the number of aggregated spike trains or auditory afferents. We reason that because spikes occur stochastically in time with a higher probability for high- than for low-intensity sounds, more afferents would be required for the nerve to faithfully encode high-frequency or low-intensity waveform features than low-frequency or high-intensity features. Deafferentation would thus degrade the encoding of these features. We further reason that due to the stochastic nature of nerve firing, the degradation would be greater in noise than in quiet. This hypothesis is tested using a vocoder. Sounds were filtered through ten adjacent frequency bands. For the signal in each band, multiple stochastically subsampled copies were obtained to roughly mimic different stochastic representations of that signal conveyed by different auditory afferents innervating a given cochlear region. These copies were then aggregated to obtain an acoustic stimulus. Tone detection and speech identification tests were performed by young, normal-hearing listeners using different numbers of stochastic samplers per frequency band in the vocoder. Results support the hypothesis that stochastic undersampling of the sound waveform, inspired by deafferentation, impairs speech perception in noise more than in quiet, consistent with auditory aging effects.

## Introduction

Auditory deafferentation is a gradual and irreversible pathology associated with aging (Makary et al., 2011) as well as to noise overexposure (Kujawa and Liberman, 2009). Although it does not manifest itself as a hearing loss in regular clinical tests, it might nevertheless have a very significant impact on auditory perception, particularly in noise (Kujawa and Liberman, 2009; Makary et al., 2011). Here, we hypothesize that deafferentation combined with the stochastic nature of action potentials degrades the quality of the neural representation of the stimulus waveform. Additionally, the effects of stochastic undersampling on auditory perception are assessed using a signal-processing tool, a vocoder.

Auditory perception probably relies on a combination of spectral and temporal information available in the acoustic stimulus. Information in the sound envelope is important for speech perception (Van Tasell et al., 1987; Rosen, 1992; Shannon et al., 1995; Smith et al., 2002), but the temporal fine structure (TFS) in the sound waveform is equally important for melody perception (Smith et al., 2002) as well as for normal speech perception in noise (Lorenzi et al., 2006). On the other hand, some spectral information is also required for envelope-based speech recognition, even in quiet (Shannon et al., 1995). Since spectral, envelope and TFS information are all important for normal auditory perception and they are all present in the stimulus waveform, this suggests that normal perception requires an appropriate neural representation of the information contained in the stimulus waveform.

The young, healthy human auditory nerve contains around 30,000 afferent fibers (Makary et al., 2011), each of which responds stochastically to the auditory stimulus. Each fiber may be thought of as conveying a stochastically digitized version of the waveform of its driving signal (the inner hair cell receptor potential) so that the aggregated response conveys a neural representation of the stimulus waveform. Alterations of a normal neural waveform representation, as occurs presumably in auditory neuropathy patients, must deteriorate auditory perception (Zeng et al., 2005). Disrupted neural waveform representations may occur by various mechanisms, including temporal desynchronization and/or deafferentation (Zeng et al., 2005). Other authors have investigated the perceptual effects of desynchronization and concluded that it impairs speech intelligibility in noise (Pichora-Fuller et al., 2007). It is uncertain, however, to what extent and which aspects of auditory perception are specifically affected by deafferentation. Some authors have conjectured that deafferentation should “decrease the robustness of stimulus coding in low signal-to-noise conditions, for example speech in noise, where spatial summation via convergence of activity from groups of neurons must be important in signal processing” [p. 14083 in Kujawa and Liberman (2009)].

The present study is motivated by the need to understand and model the perceptual consequences of deafferentation. We theorize that the stochastic nature of auditory nerve action potentials combined with a limited number of afferent fibers can limit the quality of the neural representation of the sound waveform. This limitation can affect the representation of both level and frequency information by the following two principles. First, in the absence of cochlear amplification, the *instantaneous* probability of firing of an individual auditory nerve fiber increases with increasing the instantaneous sound pressure, except, perhaps, for a saturating effect at very high pressures [e.g., Figure 4 in Heil et al. (2011)]. In other words, low-pressure sounds evoke a low probability of firing while high-pressure sounds evoke a high probability of firing. As a result, any given sound waveform feature will be more poorly represented in the response of an individual auditory fiber at low than at high pressure levels. Or put differently, sound features with low amplitudes will be more poorly represented in the spike train of an individual fiber than the features with higher amplitudes. The auditory nerve, however, contains thousands of fibers and so even though it is unlikely that each one of them will fire in response to low amplitude features, the probability of low-pressure features being preserved in the *population* response is compensated for by the large number of afferents. In other words, even though individual fibers may convey only a poor representation of low-amplitude waveform features, these features may still be faithfully represented in the population response provided there is a sufficiently large number of afferents. In other words, the “volley principle” originally proposed to explain frequency encoding (Wever, 1949) could also facilitate the representation of low-amplitude waveform features.

The second principle is that the auditory nerve action potentials occur at random along the duration of a fixed-level stimulus. Therefore, in the hypothetical absence of non-linear transient effects like adaptation, which enhances the probability of firing at the stimulus onset (Westerman and Smith, 1984), or refractoriness, a long sustained stimulus is more likely to evoke an action potential in an individual auditory nerve fiber than a shorter stimulus of the same intensity. In other words, the stochastic nature of action potentials *per se* makes it more likely that an individual auditory nerve fiber conveys sustained than transient stimuli of identical amplitudes. Since the probability of firing of an individual auditory nerve fiber is instantaneous, slowly varying periodic stimuli are more likely to be conveyed in the timing of spikes of individual afferent fibers than fast varying stimuli. In other words, an individual auditory nerve fiber is more likely to convey low than high frequency information in the spike times, even in the hypothetical absence of limited phase-locking at high frequencies attributed to low-pass filtering in the inner hair cell (Palmer and Russell, 1986). Therefore, envelope information is more likely to be represented in the timing of action potentials of individual afferents than TFS information. But again, the nerve contains thousands of fibers and so even though it is unlikely that each of them will fire in response to short features, these features may still be represented in the population response provided there is a sufficiently large number of afferents. Or conversely, a larger number of afferents is required for faithfully encoding TFS than envelope information. Indeed, this is no other than the volley principle of Wever (1949).

In summary, the probabilistic nature of action potentials *per se* combined with the number of afferents imposes a limit on the quality with which a sound waveform is represented in the population auditory nerve response. Of course, in addition to these limiting factors, the quality of the neural waveform representation is also determined by auditory nerve refractoriness, adaptation, saturation, and/or reduced phase locking at high frequencies. The point we are making, though, is that stochasticity itself, combined with a limited number of afferents, imposes a limit to information transmission. Indeed, despite these other limiting factors, a stimulus waveform is reasonably well-represented in the population nerve response over a wide range of levels (Young and Sachs, 1979; Delgutte et al., 1998) and frequencies (Heinz et al., 2001).

Here, we assess the impact of stochastic undersampling on auditory perception using a vocoder type approach inspired by auditory deafferentation. Tone detection and speech identification tests are reported for different degrees of stochastic undersampling of the stimulus waveform. We will show that stochastic undersampling impairs auditory perception in noise more than in quiet in a form broadly compatible with auditory aging.

## Materials and methods

### The vocoder

The proposed vocoder is a simple first approximation to mimic the consequences of deafferentation on information transmission due to the above described limits imposed by stochastic firing. Stimuli were filtered through a bank of ten fourth-order Butterworth filters working in parallel to roughly mimic frequency decomposition within the cochlea (the potential effects of using a different number of filters is discussed below). The cut-off frequencies of the filters were logarithmically spaced between 100 Hz and 10 kHz. Neighboring filters overlapped at their cutoff frequencies. For the signal in each band, multiple (*N*) “spike” trains were stochastically generated to roughly mimic *N* different possible representations of that signal conveyed by *N* different afferent fibers innervating a given cochlear region. Each “spike” train was obtained by sample-wise amplitude comparisons of the full-wave rectified signal with an equal-length array of random numbers. A unity amplitude “spike” was generated whenever the signal amplitude exceeded the corresponding random number. The vocoder operated in the digital domain, hence all signals had amplitudes within the range (−1, +1). For this reason, random numbers were chosen to have values *uniformly* distributed between 0 and 1. The resulting *N* “spike” trains per frequency band were aggregated into a single “spike” train using a sample-wise logical OR function; that is, unity amplitude “spikes” occurred in the aggregated response whenever a “spike” occurred in any of the *N* available spike trains. An acoustic version of the aggregated “spike” train was then obtained by sample-wise multiplication of the train in question with the original signal in each band. An OR function was used rather than the mean so that for a sufficiently large *N*, the reconstructed acoustic signal converged to the original one, a reasonable prerequisite. Finally, the reconstructed signal from each frequency band was filtered through its corresponding Butterworth filter to maintain the spectral content in the band (i.e., to filter out distortion or energy splatter), and the ten resulting signals, one per band, were sample-wise added to obtain a vocoded stimulus. A detailed explanation of the stochastic sampling mechanism and its consequences on information transmission is provided in the Appendix.

### Experiments

#### Approach

Our aim was to test the hypothesis that stochastic undersampling of the sound waveform inspired by deafferentation decreases the robustness of stimulus encoding in low signal-to-noise conditions, as suggested elsewhere (Kujawa and Liberman, 2009). We measured speech reception thresholds (SRTs) and pure tone detection thresholds in quiet and in fixed-level noise using vocoded stimuli with a large (*N* = 300) and a small (*N* = 10) number of stochastic samplers per frequency channel. The control condition consisted of using stimuli vocoded without the stochastic sampling stage. Our hypothesis was that performance would be comparable for *N* = 300 and the control conditions, but would deteriorate for the *N* = 10, suggesting that normal performance requires a sufficiently large number of stochastic samplers or, conversely, that stochastic undersampling deteriorates performance. Decreasing *N* not only degrades the waveform but also reduces the stimulus energy in the vocoded stimuli and hence audibility (i.e., in the extreme, setting *N* = 0 would mimic a hypothetical case of a “dead” cochlea with no functional afferents). To investigate the effects of stochastic undersampling on perception aside from its effects on overall loudness/audibility, vocoded stimuli were equated for rms energy throughout conditions (see the Discussion). We also tested our main hypothesis analytically, by measuring the degree of temporal correlation between a control signal (a tone or a word) in quiet and the vocoded stimuli for various signal-to-noise ratios (SNRs) and number of stochastic samplers (*N*) per frequency channel (see below).

Human experiments conformed to the requirements of the Ethical Review Board of the University of Salamanca.

#### Pure tone detection thresholds

Detection thresholds in quiet and in fixed-level white noise were measured for pure tones at audiometric frequencies from 250 Hz to 8 kHz in octave steps. Pure tones had durations of 100 ms, including 5-ms raised-cosine onset and offset ramps. The total noise duration was 300 ms, including 10-ms onset and offset ramps. The noise started 100 ms before the tone onset and ended 100 ms after the tone offset. The noise level was fixed at 65 dB SPL. Threshold was defined as the level at which the tones were detected on 70% of the occasions that they were presented. A two-down, one-up adaptive procedure was used to measure threshold (Levitt, 1971). Three threshold estimates were obtained per frequency and the mean was taken as the detection threshold. If the standard deviation of the three estimates exceeded 6 dB, a fourth estimated was obtained and included in the mean.

#### Speech identification test

The Castilian-Spanish version of the hearing in noise test (HINT) was used to measure SRTs in diotic, speech-shaped noise with a fixed level of 65 dB SPL (Nilsson et al., 1994; Huarte, 2008). SRT was defined as the speech-to-noise ratio (SNR), in decibels, required for listeners to correctly identify 50% of the sentences they were presented with. Three SRTs estimates were measured per condition, the mean of which was taken as the SRT.

Speech identification was measured also in quiet for a fixed speech SPL equal to the individual value at which each listener achieved 50% correct in noise. Twenty HINT sentences were presented and the number of correctly identified sentences was noted. No sentence was repeated throughout conditions.

#### Listeners

Twelve female, young listeners with normal hearing participated in the experiments. Their ages ranged from 22 to 27 years, with a mean age of 23.4 years. All of them had audiometric thresholds less than 20 dB HL at frequencies from 500 to 8000 Hz (ANSI 1996) and self-reported no history of hearing impairment. Six subjects were tested in their left ears and six were tested in their right ears. All subjects participated in the HINT test, ten subjects participated in the pure tone-in-noise detection threshold test, and five subjects participated in the speech identification and pure-tone detection tests in quiet. Subjects were volunteers and were not paid for their services. They all signed an informed consent.

#### Stimuli and apparatus

Stimuli were generated digitally (sampling frequency was 22050 Hz) and vocoded prior to presentation to the listeners. Vocoded stimuli were digital-to-analogue converted using a RME Fireface 400 sound card with a resolution of 24 bits, and presented through circumaural Sennheiser HD580 headphones. Subjects sat in a double-wall sound booth during testing.

#### Statistical analysis

Unless otherwise stated, mean results across conditions were compared using two-tailed, paired Student's *t*-tests and the null hypothesis was rejected at the *p* < 0.05 significance level.

## Results

### An example vocoded stimulus

**Figure 1:**
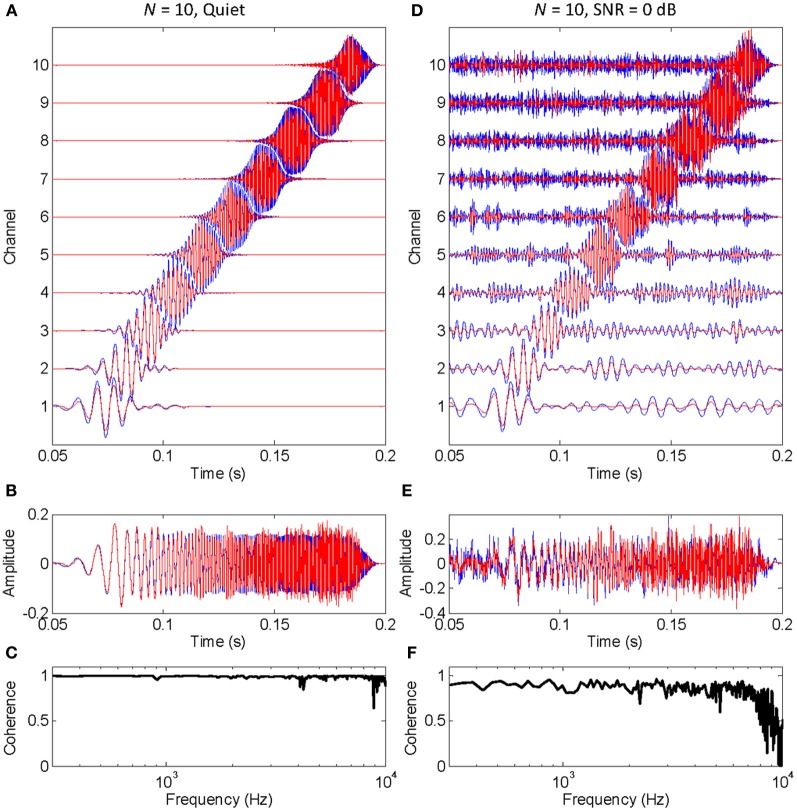
**An example of how stochastic undersampling degrades the stimulus waveform**. In this example, 10 stochastic samplers were used per frequency band (or channel). The stimulus was a chirp from 20 Hz to 12 kHz with constant peak amplitude of 0.1, presented in quiet (left panels) and in 0 dB SNR pink noise (right panels). **(A,D)** Vocoded (red) and control (blue) signals at the output of each frequency channel. Channel number is proportional to channel frequency (i.e., channel 1 is the lowest-frequency channel). **(B,E)** Vocoded (red) and control stimuli (blue) that result from sample-wise addition of the signals in each channel. **(C,F)** Cross-spectral coherence between the vocoded and control stimuli.

**Figure 2:**
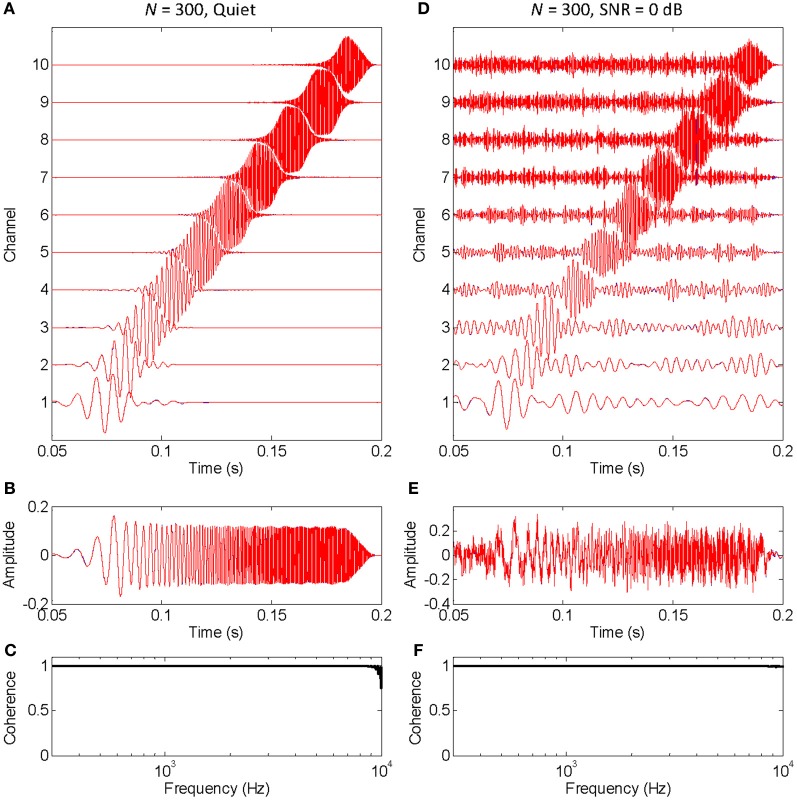
**An example that the vocoded stimulus converges to the control stimulus when a large number of stochastic samplers per frequency band, or channels, is considered**. This is the same example as in Figure 1, except that the chirp was vocoded using 300 instead of 10 stochastic samplers per channel. The panels are as in Figure 1. Note that no blue traces are seen because the red traces overlap perfectly with the blue traces, a sign that the vocoded signals converged to the control signals. Note, also, that spectral coherence was optimal (equal to unity) across virtually the full frequency range both in quiet and noise, a demonstration that the vocoded and control stimuli had virtually identical spectra.

The functioning of the vocoder for a small (*N* = 10) and large (*N* = 300) number of stochastic samplers per frequency band, or channel, is illustrated in Figures 1, 2, respectively. The stimulus was a chirp (constant sweep rate in log-Hz from 20 Hz to 12 kHz) with constant peak amplitude of 0.1 and rise/fall times of 20 ms (sampling frequency of 44.1 kHz). This stimulus was chosen to better illustrate that the chosen stochastic sampling method is more likely to preserve slow- than the fast-varying waveform features; in this case, the earlier, lower frequency portions of the chirp waveform over the later, higher frequency portions of the chirp waveform. The left and right columns of each figure illustrate results for the chirp in quiet and in 0 dB SNR pink noise, respectively. The top panels in each figure (panels **A** and **D**) illustrate the output signals from each frequency channel in the vocoder; blue traces show control signals (i.e., vocoded without the stochastic samplers) while red traces show signals obtained by sample-wise aggregation of the *N* stochastically sampled control signals. The mid panels in each figure (**B** and **E**) illustrate control (blue) and vocoded (red) stimuli. Lastly, the bottom panels in each figure (**C** and **F**) illustrate the magnitude cross-spectral coherence between control and vocoded stimuli (i.e., between the blue and red traces in panels **B** and **E**). The magnitude cross-spectral coherence is a measure of the temporal correlation between two signals across their different spectral components.

The left panels in Figure 1 show that stochastic undersampling (i.e., using *N* = 10) slightly deteriorates the chirp waveform in quiet and that the deterioration is greater for the faster varying (higher frequency) portion of the stimulus waveform than for the slowly varying (lower frequency) portions of the stimulus waveform. This is shown by the comparatively better overlap between the blue and red traces in the lower than in the higher frequency channels of the vocoder (Figure 1A), or at the beginning than at the end of the vocoded stimulus (Figure 1B). It is also demonstrated by the nearly perfect cross-spectral coherence at low frequencies compared to the “noisier” coherence values at higher frequencies (Figure 1C).

The left and right panels in Figure 1 are for an identical chirp in quiet and noise, respectively. A comparison between the vocoded stimuli in these two cases shows that the negative effects of stochastic undersampling are considerably greater in noise than in quiet, particularly for fast-varying waveform features. Note, for example, that the cross-spectral coherence at high frequencies is overall smaller (and “noisier”) in noise (Figure 1F) than in quiet (Figure 1C).

Figure 2 is identical to Figure 1 except that it was obtained using a larger number of stochastic samplers per frequency band (*N* = 300). There is virtually no sign of blue traces (control signals) because they are underneath the red traces (vocoded signals). Furthermore, the cross-spectral coherence between vocoded and control stimuli is optimal (equal to unity) across virtually all frequencies, both in quiet (Figure 2C) and in noise (Figure 2F). Altogether, this shows that the chosen stochastic sampling mechanism does not degrade the vocoded stimulus waveform either in quiet or in noise when a sufficiently large number of stochastic samplers is used.

### Pure tone detection thresholds

**Figure 3:**
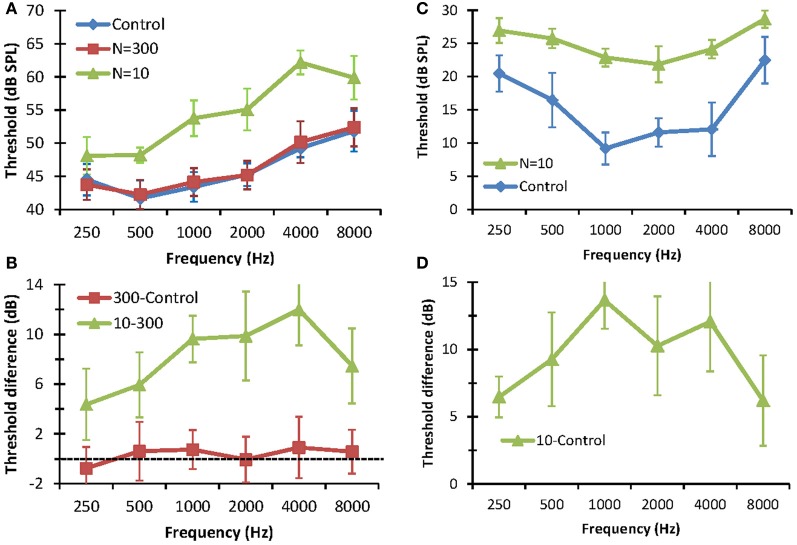
**Effect of reducing the number stochastic samplers (*N*) on the detection thresholds of pure tones in noise (left panels) and in quiet (right panels). (A)** Detection thresholds in fixed-level (65 dB SPL) white noise. Each series is for a different condition. **(B)** Differences between detection thresholds in noise measured in different conditions, as indicated by the legend. **(C)**. Detection thresholds in quiet. Each series is for a different condition. **(D)** Differences between detection thresholds in quiet. In all panels, error bars illustrate one standard deviation.

The effect of reducing the number stochastic copies (*N*) on the detection thresholds of pure tones is shown in Figure 3. The left panels show detection thresholds in *noise*. In the control condition (no stochastic sampling), thresholds increased with increasing tone frequency (Figure 3A). This result is consistent with the idea that the critical band increases with increasing signal frequency as result of increased filter bandwidth (Moore, 2007). No statistically significant differences were observed between detection thresholds for *N* = 300 and the control conditions. This shows that using 300 stochastic samplers per frequency channel (3000 samplers in total) is sufficient to produce normal thresholds. Detection thresholds for *N* = 10, by contrast, were significantly higher than those for *N* = 300 or the control conditions. Furthermore, there was a trend for the difference between thresholds for *N* = 10 and *N* = 300 to increase with increasing frequency, from 3 dB at 250 Hz to 12 dB at 4000 Hz (Figure 3B). Altogether, these results are consistent with the hypothesis that reducing the number of stochastic samplers at the output of each frequency channel degrades performance in noise and that degradation affects higher frequencies (i.e., faster varying sounds) more than lower frequencies. Note that this degradation is not due to reduced stimulus rms energy, as vocoded and control stimuli were equated for rms energy. Instead it was due to differences in stimulus energy distribution along time; i.e., to changes in the stimulus waveforms. Note, also, the threshold difference was lower at 8 kHz (7.47 dB) than at 4 kHz (12 dB), suggesting that reducing *N* affected the mid-frequencies more than the highest frequency tested. The reason for this result is uncertain.

The right panels of Figure 3 show the effects of stochastic undersampling on pure tone detection thresholds in *quiet*. Figure 3C shows mean pure tone thresholds in quiet for control stimuli and for vocoded stimuli with *N* = 10. Unlike thresholds in noise, which increased with increasing frequency (Figure 3A), thresholds in quiet varied with frequency according to the typical shape of a human audiogram (in dB SPL units) [e.g., Figure 2.1 in Moore (2007)]. Thresholds for *N* = 10 were significantly higher than for the control condition (Figure 3C). As in noise, this increase was not due to differences in rms energy between vocoded and control stimuli but to differences in their energy distributions along time. For example, in the extreme case, a very low-amplitude pure tone would evoke only one “spike” along the whole stimulus waveform in the vocoder channel corresponding to the frequency of the tone. That “spike” would be filtered through the channel back-end filter, effectively generating a vocoded stimulus equal to the impulse response of that filter. As a result, this vocoded stimulus would broadly preserve the frequency content of the control stimulus but it would be shorter and would have a higher peak amplitude than the control stimulus. Interestingly, though, stochastic undersampling raised thresholds in quiet by less than 15 dB, which is within the range typically regarded as “normal hearing” [e.g., p. 43 in Moore (2007)]. In other words, stochastic undersampling raised pure tone thresholds in quiet but did not produce a clinical hearing loss. As in noise, the threshold increase in quiet was greater for mid- than for low- or high-frequencies (Figure 3D). The reason for this result is uncertain.

### Speech-in-noise identification test

**Figure 4:**
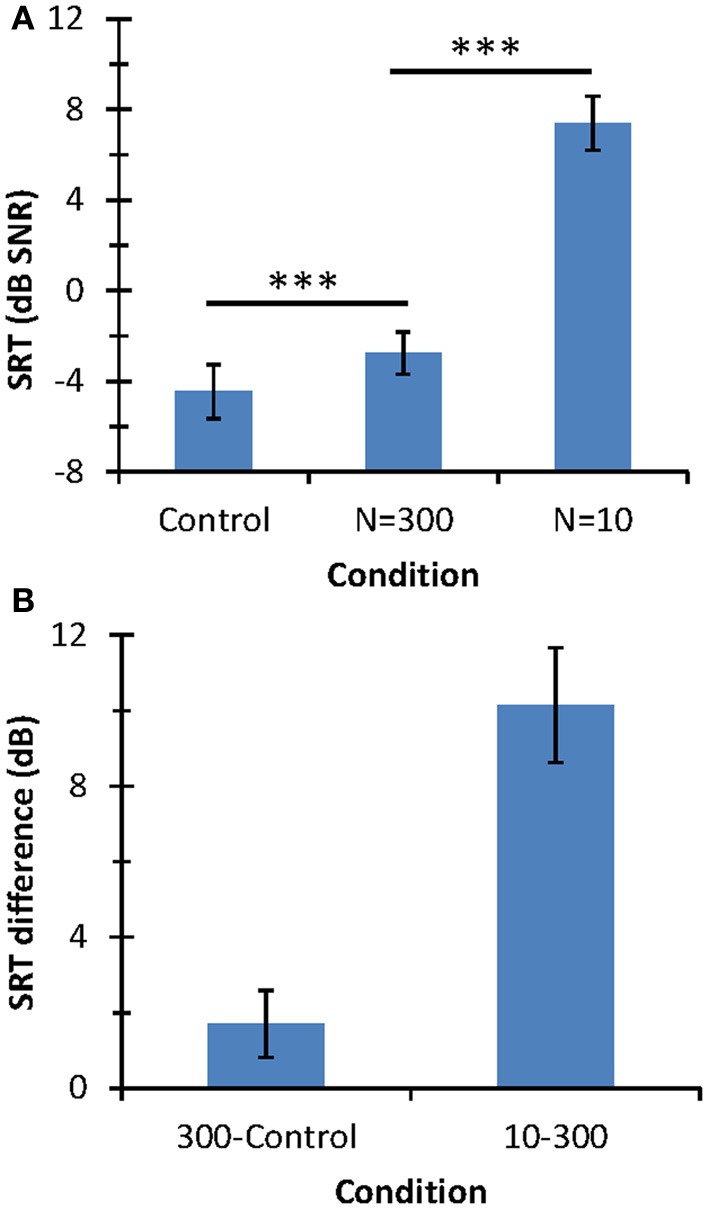
**Speech-in-noise identification results. (A)** Mean SRT (in dB SNR). Horizontal lines indicate the statistical comparisons made and three asterisks indicate a level of significance of *p* < 0.001. **(B)** SRT differences across conditions. In all panels, error bars illustrate one standard deviation.

Figure 4 illustrates the results of the speech-in-noise identification test. The mean SRT for the control condition was −4.46 dB SNR (*SD* = 1.2 dB). This value is slightly lower but still comparable to the normative value for a corresponding condition [mean = −3.6 dB SNR; *SD* = 1.2 dB (Huarte, 2008)]. The present SRT may be slightly lower than the normative value because our listeners were younger (22–27 years) and had better audiometric thresholds (<20 dB HL) than those used to obtain normative values (20–50 years and 25 dB HL).

The mean SRT was lower for the control condition (−4.46 dB SNR) than for *N* = 300 (−2.76 dB SNR) or *N* = 10 (+7.38 dB SNR). Although mean SRTs were statistically different across the three conditions (as depicted by the asterisks in Figure 4A), SRT was much higher for *N* = 10 than for the other two conditions; indeed, SRTs for the control condition and the *N* = 300 differed only by 1.7 dB while the SRT increased by 10.15 dB with decreasing *N* from 300 to 10. In summary, speech-in-noise identification with respect to the control condition degraded significantly more when using 10 than 300 stochastic samplers per frequency channel.

In quiet, participants correctly identified ~100% of the HINT sentences when they were presented at the SPL at which listeners only achieved 50% correct performance in noise, even for *N* = 10. This is not to say, however, that the speech sounded natural in quiet when vocoded using *N* = 10; indeed, some degradation could be perceived in quiet. What is important, though, is that for a fixed signal level, the degradation in question deteriorated intelligibility in noise more than in quiet.

### Analytical results

The above experimental results demonstrate the importance of a sufficiently large number of stochastic samplers for normal speech perception and that a stochastically degraded waveform can lead to poorer detectability and intelligibility in noise, depending on the used number of stochastic samplers per frequency channel. For practical reasons, the reported experimental results are for a limited number of conditions. To assess the importance of stochastic undersampling on perception for an extended number of conditions, we quantified the degree of temporal correlation between a vocoded stimulus (speech + noise) and the target speech in quiet in the control condition. The assumption behind this approach was that speech intelligibility in noise depends on the temporal similarity between the vocoded noisy stimulus and an optimal representation of the target speech in quiet. Recall that the chosen algorithm of stochastic sampling was intended to account for the physiological property that large amplitude waveform features are more likely to be represented than low amplitude features in the response of individual afferents. To assess the interaction of speech level with *N* and SNR, the degree of temporal correlation was assessed for different speech rms levels.

**Figure 5:**
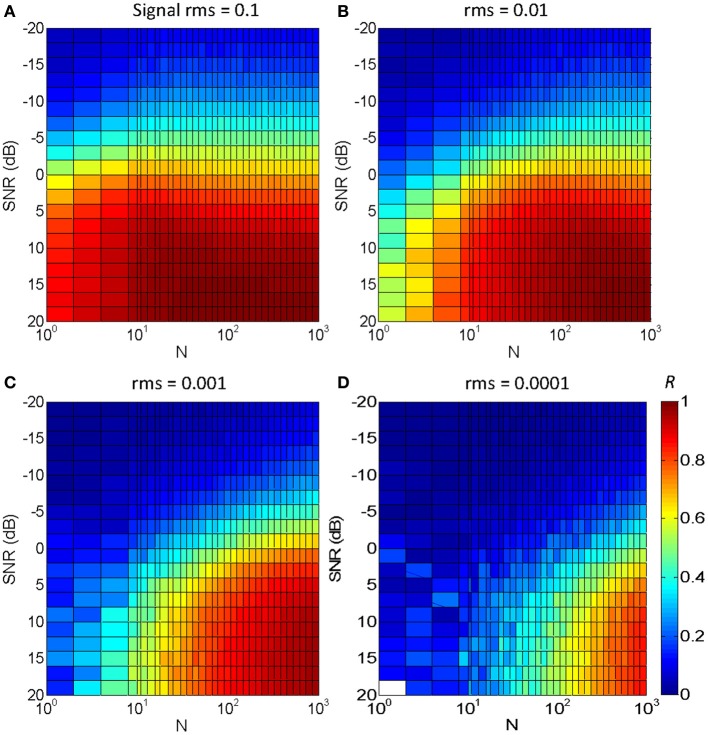
**Temporal correlation for different SNR and *N* between a vocoded word-in-noise and the control word in quiet**. The word was “*mujer*,” the Castilian Spanish for “woman.” Color illustrates maximum correlation (*R*) from 0 (dark blue) to 1 (dark red), as shown by the color map in panel **(D)**. Each panel is for a different speech rms level: **(A)** 0.1; **(B)** 0.01; **(C)** 0.001; **(D)** 0.0001.

Figure 5 illustrates the results for an utterance of the word “*mujer*” (the Spanish word for “woman”) in Gaussian white noise. Each panel is for a different signal rms level, with the four panels covering a 60 dB range. The color gradient illustrates the degree of temporal correlation (*R*): red indicates a high correlation (*R* ~ 1) while blue indicates a low correlation (*R* ~ 0), as shown in the color map of Figure 5D. The main results may be summarized as follows:

1. Optimal temporal correlation (*R* = 1) was obtained in optimal conditions (SNR = 20 dB and *N* = 1000). This shows that the vocoded stimulus converged to the original speech signal in quiet, as intended (see also Figure 2).
2. For the largest *N* (=1000) and an intermediate speech level (rms = 0.01, Figure 5B), temporal correlation decreased from 1 to 0 with decreasing SNR from 20 to −20 dB. This decrease is due to the masking effect of the noise rather than to the stochastic sampling and could be thought of as reflecting the typical percept that speech intelligibility decreases with decreasing SNR.
3. For a fixed *N* and SNR, temporal correlation decreased with decreasing speech level. This is due to a reduced probability of “firing” at very low levels. It implies that even for a large *N*, the signal waveform is more poorly represented at low signal levels than at high levels, as intended. Or, conversely, even for small *N*, the signal is reasonably well-represented at high levels.
4. For a fixed speech level and SNR, temporal correlation decreases with decreasing *N*. This shows that stochastic (under)sampling deteriorates the signal waveform and is qualitatively consistent with the present experimental results (Figure 4).
5. For a fixed speech level, say rms = 0.001 (Figure 5C), reducing *N* decreases temporal correlation in noise more than in quiet. In other words, to obtain a fixed correlation, as would presumably be required to achieve constant speech intelligibility, a larger *N* would be required in noise than in quiet.
6. For a fixed speech level, say rms = 0.001 (Figure 5C), the SNR required to achieve a fixed correlation depends on *N* to a certain extent. For example, to achieve a fixed correlation *R* = 0.5 (green color) the approximate SNR would be 12.5 dB for *N* = 10 and −3 dB for *N* = 300. This result is qualitatively consistent with the present experimental results for the speech intelligibility test. That is, assuming that a fixed temporal correlation (say *R* = 0.5) is required for SRT (i.e., to achieve a fixed 50% correct speech identification), this suggests that the SRT would be higher for *N* = 10 than for *N* = 300, consistent with the experimental results (Figure 4).

#### The effect of the number of filters

**Figure 6:**
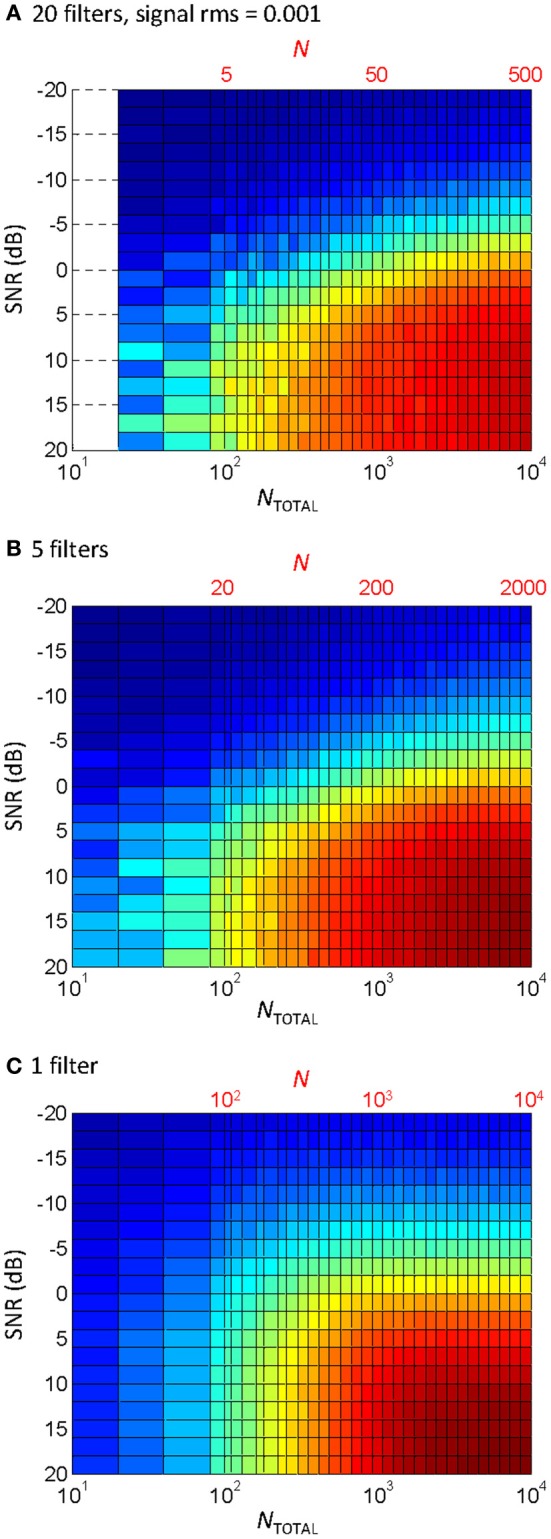
**The effect of the number of filters and *N* on the temporal correlation between a vocoded word-in-noise and the control word in quiet**. This is the same analysis as in Figure 5C, except that different number of filters were used in the vocoder: **(A)** 20 filters; **(B)** 5 filters; **(C)** 1 filter. Note that each panel has two abscissa scales: the top scale (in red font) is the number of samplers per channel (*N*); the bottom scale denotes the total number of samplers (*N*_TOTAL_) obtained as the number of filters times *N*. Note also that *N* varied across the three panels while *N*_TOTAL_ remained identical. Note also that *N*_TOTAL_ was identical as in Figure 5.

Ten filters were used in the vocoder to compromise between computational speed (the behavioral experiments involved processing stimuli while the task was running) and the known need for a certain amount of spectral information for speech perception (e.g., Shannon et al., 1995). Physiological cochlear filters, however, are not fixed in number but rather they are distributed continuously along the cochlear partition (e.g., Greenwood, 1990). Therefore, one may wonder whether the pattern of results would be different for a different number of filters. To address this question, the correlation analysis shown in Figure 5C was repeated using different numbers of filters. The results are shown in Figure 6. Note that the bottom abscissa in Figure 6 illustrates the total number of samplers in the vocoder (*N*_TOTAL_ = *N* × number of filters) rather than *N*, as shown in Figure 5, and that *N*_TOTAL_ is identical for all the panels in Figures 5, 6. Clearly, the pattern of results was similar for 20 filters (Figure 6A) and five filters (Figure 6B), and so presumably using more than ten filters, as we used here (Figure 5C), would have hardly changed the behavioral results. If anything, a larger *N* increases information transmission per frequency band (see the Appendix), which explains the slight trend for higher correlations (higher *R*) for five (Figure 6B) than for 20 filters (Figure 6A) for any given condition (as defined by SNR and *N*_TOTAL_). On the other hand, the spread of information across several filters allows for a reduction of *N* without significantly affecting the pattern of results. Figure 6C illustrates that the effect of reducing *N* for a vocoder with one single filter (note that *N* = *N*_TOTAL_ in this case). As expected, the degree of temporal correlation decreases more rapidly with decreasing *N* in this case than when five or more filters are used. In other words, reducing *N*_TOTAL_ beyond a certain value reduces the degree of temporal correlation more rapidly when the waveform information is all within a single frequency channel than when it is distributed across a number of channels.

## Discussion

The proposed vocoder is a highly simplified tool inspired by auditory deafferentation to explore the effects of stochastic undersampling on auditory perception. It serves to test the hypothesis, inspired by the volley principle, that deafferentation combined with the stochastic nature of individual afferent fiber responses degrades the neural representation of the stimulus waveform by degradation of low-amplitude and high-frequency features.

We have experimentally and analytically shown that stochastic degradation of the stimulus waveform deteriorates speech perception in noise more than in quiet. It is well-known that the defects of typical stochastic sampling methods can be characterized as noise (Dippé and Wold, 1985). The present physiologically inspired algorithm adds a level-dependency to stochastic sampling, effectively leading to represent undersampled high frequencies and low amplitudes as noise (see the Appendix). For a stimulus consisting of a signal embedded in noise, the degradation would similarly affect the signal and the noise. Degradation of the noise would effectively generate a different noise, but noise nonetheless, while degradation of the signal would turn part of the signal into noise. The net effect is a reduction of the SNR, as evidenced by the present results. The present results thus support the hypothesis of Kujawa and Liberman (2009) that deafferentation probably degrades the neural representation of speech in noise. It is unclear, however, how the mechanism proposed and explored here relates to their proposed mechanism of “spatial summation via convergence of activity from groups of neurons.”

The chosen stochastic sampling algorithm is such that the probability of “firing” increases gradually with increasing instantaneous pressure. In other words, it could be thought of as mimicking a non-saturating rate-level function of the so-called “straight” type, which is characteristic of fibers with medium and low spontaneous rates [Figure 2F in Winter et al. (1990)]. Incidentally, it has been shown during the course of the present work that neuropathic noise causes precisely a loss of this type of fibers (Furman et al., 2013). A more realistic vocoder might be constructed by using a different stochastic sampler that includes refractoriness and saturation, or concurrent stochastic samplers with different rate thresholds and rate-level functions in an attempt to mimic more closely the diversity of response characteristics of real nerve fibers (Liberman, 1978; Winter et al., 1990). These factors would probably introduce additional restrictions that would further degrade the waveform representation as a result of reducing *N*.

The chosen stochastic sampling algorithm gives high-pressure waveform features a higher probability of being preserved in the vocoded stimulus than low-pressure features. As a result, the quality of the vocoded waveform degrades with decreasing sound level or decreasing *N*, the number of stochastic samplers. This suggests that the stochastic undersampling here attributed to deafferentation might have a greater impact at low than at high levels (compare Figure 5A with Figure 5D). Incidentally, it is tempting to conjecture that nature provided mammals with a comparatively larger number of nerve fibers with low than with high rate thresholds (Liberman, 1978; Winter et al., 1990) to minimize the potential impact of deafferentation or to faithfully encode low-intensity sound waveform features. Likewise, the present time-linear (or adaptation-free) stochastic sampling algorithm degrades high-frequencies, and hence waveform transients, more than low-frequencies or sustained waveform features (see the Appendix). Hence, it is also temping to conjecture that the greater probability of auditory nerve fibers to fire at the stimulus onset than during the steady state (Westerman and Smith, 1984) serves to minimize the impact of deafferentation and/or to more faithfully encode fast transient waveform features than would otherwise be poorly represented due to the stochastic nature of action potentials.

Our choice to equate the rms amplitude of vocoded and control stimuli was intended to reveal the potential effects of altering temporal information (Pichora-Fuller et al., 2007) as well as the natural balance between low- and high-amplitude waveform features due to stochastic undersampling independently from reduced overall loudness or audibility. Deafferentation, however, probably reduces overall nerve firing, which may in turn reduce overall loudness. Therefore, by equalizing rms levels, we have disregarded the potential effects of deafferentation on overall loudness. Furthermore, rms level equalization may have caused vocoded waveforms to be “peakier” than the control waveforms (e.g., Figure 1B). While back-end filtering guarantees comparable spectra for control and vocoded stimuli, it is conceivable that the “peakier” vocoded stimuli may have been subject to different compression regimes in the listeners' cochleae than the control stimuli. This, in turn, may have caused different amounts of cochlear distortion for vocoded and control stimuli. It is uncertain to what extent, if anything at all, potential differences in cochlear distortion caused by vocoded and control stimuli may have affected or can contribute to explain the present behavioral results.

It must be stressed that, while inspired by deafferentation, the proposed vocoder is far from an accurate physiological model of deafferentation and it is uncertain that vocoded stimuli would evoke physiological responses in a fully afferented auditory nerve comparable to the responses evoked by control stimuli in a deafferented nerve. Instead, the vocoder is a tool to model the reduction of *information* associated to stochastic undersampling, as explained in the Appendix. In other words, it has been designed to model the consequences of stochastic reduction of information on auditory *perception*. As such, validation should be made against experimental behavioral data for patients known to suffer from auditory deafferentation. This is difficult for we lack reliable tests to diagnose auditory deafferentation and so we lack data for patients known to suffer from this specific disease. Deafferentation, however, is a particular form of auditory neuropathy (Zeng et al., 2005; Zeng and Liu, 2006). Furthermore, it comes with age (Makary et al., 2011) and probably occurs in normal-hearing listeners who suffer frequent temporary threshold shifts (Kujawa and Liberman, 2009). Therefore, the vocoder may be validated by comparing the perception it evokes in normal-hearing listeners with data for (*a*) aged listeners with normal audiometric thresholds, or (*b*) young listeners with frequent temporary threshold shifts, or (*c*) auditory neuropathy patients. In this regard, speech-in-noise identification is poorer for elderly listeners with audiometrically normal hearing than for young normal-hearing listeners (Pichora-Fuller et al., 2007; Fullgrabe et al., 2011). This is qualitatively consistent with the present experimental result that stochastic undersampling increases the SRT (Figure 4) disproportionally more than the audiometric loss (Figure 3). Also, masked detection thresholds are higher for auditory neuropathy patients than for normal-hearing patients [Figures 8, 9 of Zeng et al. (2005)], which is also consistent with the present observation that masked detection thresholds increase with decreasing *N* (Figure 3B). Lastly, the present results and vocoder are also consistent with the idea that elderly listeners with normal hearing suffer reduced temporal precision of speech encoding (Anderson et al., 2012).

In summary, although for a limited number of conditions, the present results support the proposed vocoder as a tool to explore the consequences of auditory deafferentation and aging on auditory perception. Further work is required to assess its validity at mimicking other age- and/or neuropathy-specific auditory deficits, particularly impaired temporal gap detection (e.g., Schneider and Hamstra, 1999; Zeng et al., 2005; Pichora-Fuller et al., 2006).

## Conclusions

1. The stochastic nature of action potentials *per se* likely imposes a limit to information encoding in the auditory nerve.
2. Because of the stochastic nature of action potentials, deafferentation likely degrades the neural encoding of low-intensity and high-frequency waveform features.
3. Stochastic undersampling of the sound waveform, as inspired by deafferentation, impairs auditory perception in noise more than in quiet.
4. Stochastic undersampling of a sound waveform following simple physiological level-dependent rules degrades auditory perception in a form broadly compatible with auditory aging.

### Conflict of interest statement

The authors declare that the research was conducted in the absence of any commercial or financial relationships that could be construed as a potential conflict of interest.

## Figures and Tables

**Figure A1:**
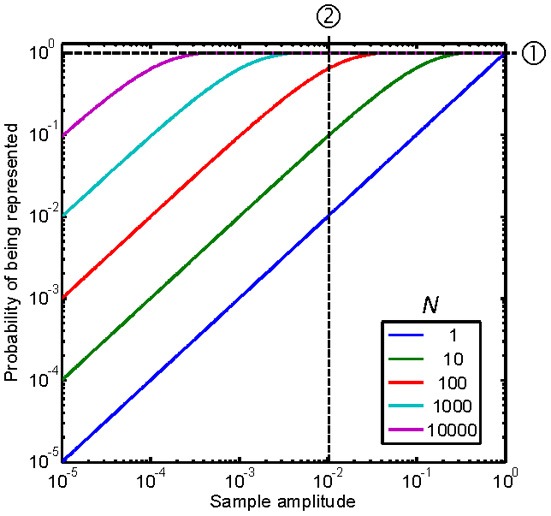
**Probability of representing a sample of the original signal in the stochastically subsampled version as a function of the sample amplitude and the number of stochastic samplers, *N***. Each curve is for a different *N*, as indicated by the legend. See text for details.

## Appendix. The stochastic sampling algorithm in detail

The stochastic sampling algorithm was designed to account for the principles explained in the Introduction rather than to closely mimic auditory physiology. Specifically, it was designed to meet two prerequisites: (1) that the instantaneous firing probability of an individual auditory nerve fiber is proportional to the instantaneous sound pressure; (2) that for a sufficiently large number of stochastic samplers, *N*, the reconstructed waveform at the output of the stochastic sampling stage should converge (i.e., be identical) to the original one. The latter was intended to account for the fact that normal perception is determined by a normal (undegraded) stimulus waveform.

Let *x*[*i*] be the digital input sound waveform to the vocoder. Because it was a digital signal, its instantaneous amplitude was always within the interval (−1, +1). This is not to say that *x*[*i*] was always normalized to span the full amplitude range; instead, its actual amplitude range depended upon the desired rms level of the stimulus. Let *y*_*k*_[*i*] be the corresponding output waveform in the *k*-th frequency channel. The stochastic algorithm was applied independently on the output signal of each frequency channel. Therefore, for convenience, in what follows we will simplify the notation by omitting the *k* subscript.

For each *y*[*i*], multiple (*N*) unity-amplitude “spike” trains, *s*_*j*_[*i*] with *j* = 1…*N*, were stochastically generated as follows:

(A1) $$s_{j}\left[i\right]=\{\begin{array}{c} 1,\text{ if}\left|y\left[i\right]\right|\geq r_{j}\left[i\right]\\ 0,\text{ otherwise} \end{array},$$

where |*y*[*i*]| denotes the absolute value of *y*[*i*], and *r*_*j*_[*i*] denotes an array of random numbers uniformly distributed between 0 and 1. Note that a different random number array was used for each stochastic sampler (see the example in Table A1), and that |*y*[*i*]| is equivalent to full-wave rectification (FWR) of *y*[*i*].

**Table A1:** **A highly simplified, artificial example to illustrate the operation of the stochastic (under)sampling algorithm for a signal *y*[*i*] with five samples (*i* = 1 … 5) and three stochastic samplers (*N* = 3)**.

**Sample number**		***i***	**1**	**2**	**3**	**4**	**5**
Input signal		*y*[*i*]	0.0156	−0.0011	0.5131	−0.3025	0.0001
FWR input signal		|*y*[*i*]|	0.0156	0.0011	0.5131	0.3025	0.0001
Random-number series	1	*r*_1_[*i*]	0.5882	0.5303	0.1463	0.8907	0.5959
	2	*r*_2_[*i*]	0.0267	0.1484	0.6542	0.7647	0.9968
	3	*r*_3_[*i*]	0.9556	0.5331	0.2005	0.2968	0.0873
Binary “spike” trains (Equation A1)	1	*s*_1_[*i*]	0	0	1	0	0
	2	*s*_2_[*i*]	0	0	0	0	0
	3	*s*_3_[*i*]	0	0	1	1	0
OR-aggregated “spike” train (Equation A3)		*S*[*i*]	0	0	1	1	0
Undersampled signal (Equation A2)		*z*[*i*]	0	0	0.5131	−0.3025	0

Note that a unity-amplitude spike occurs when the corresponding random number is lower than the corresponding sample value y[i]; e.g., s_1_[3] = 1 because y[3] ≥ r_1_[3]. FWR, full-wave rectified.

The reconstructed signal at the output of the stochastic sampling stage, *z*[*i*], was calculated as:

(A2) z[i]=y[i]·S[i],

where the dot (.) denotes sample-wise multiplication, and *S*[*i*] is the aggregated spike train, which was obtained as follows:

(A3) $$S[i]=\vee_{j=1}^{N}\left(s_{j}[i]\right)$$

where V denotes the logical OR, or disjunction, operator.

Table A1 illustrates a highly simplified example of the stochastic sampling algorithm for a signal *y*[*i*] with only five samples (*i* = 1…5) and three stochastic samplers (*N* = 3). In this example, only samples #3 and #4 of the original signal, *y*[*i*], remained in the stochastically subsampled, or reconstructed, signal *z*[*i*].

Several considerations are in order. First, we could have obtained a reconstructed signal, *z*[*i*], by sample-wise summation of the *N* “spike” trains as follows:

(A4) $$z[i]=sign(y[i])\cdot \sum_{j=1}^{N}s_{j}[i],$$

In this case, *z*[*i*] would resemble a post-stimulus time histogram conceptually similar to those used in physiological analyses (except for the *sign* which would be necessary for the reconstructed signal to preserve the polarity of the original one). This approach, however, would have produced a noisier *z*[*i*], which would have made it impossible to meet the second prerequisite listed above with a limited *N*. Note, nonetheless, that Equations A2 and A4 are mathematically equivalent when *N* tends to infinity and after proper normalization of Equation A4 to max(|*y*[*i*]|). Therefore, our approach is conceptually reasonable and not far from physiological procedures.

The second consideration is that our approach allows obtaining an analytic expression for the probability, *p*[*i*], of preserving a sample of the original signal in the reconstructed signal, i.e., for the probability that *S*[*i*] = 1. By virtue of using uniformly distributed random numbers (Equation A1), the probability of a stochastic sampler “firing” at the *i*-th epoch is equal to the absolute signal amplitude at that epoch, |*y*[*i*]|. Hence, (1 − |*y*[*i*]|) is the probability of the sampler *not* “firing,” and (1 − |*y*[*i*]|)^*N*^ the probability of *none* of the *N* available samplers “firing” at that epoch. Therefore, the probability of *at least one* sampler firing at the *i*-th epoch equals:

(A5) $$p[i]=1-{\left(1-\left|y\left[i\right]\right|\right)}^{N}.$$

Figure A1 illustrates this probability as a function of the sample amplitude, with *N* as a parameter. Note that for any given sample amplitude, *y*[*i*], the probability increases with increasing *N*, as illustrated by the vertical line ➁ in the figure.

A third consideration is that perfect reconstruction of the original signal would require a “spike” occurring in the aggregated spike train at each and every epoch. Since the probability of a “spike” occurring at one epoch is independent of the probability at any other epoch, the probability of perfect reconstruction, *p*(*z* = *y*), would be the product probability across all the samples, *N*_S_, in the original signal:

(A6) $$p(z=y)=\prod_{i=1}^{N_{s}}p[i]\;\forall y\left[i\right]\neq 0,$$

Given that 0 ≤ *p*[*i*] ≤ 1, Equation A6 demonstrates that the probability of *perfect* reconstruction rapidly tends to zero with increasing *N*_S_ (i.e., missing only one sample would be sufficient not to achieve perfect reconstruction).

This is not to say, however, that the *information* present in the reconstructed signal is zero for all stimuli, as evidenced by the present results. Nyquist-Shannon's sampling theorem states that a band-limited function can be perfectly reconstructed from an infinite sequence of samples if the band-limit, *F*_max_, is smaller than half the sampling rate, *F*_S_ (in samples per second). The reconstructed signal may be understood as a stochastically subsampled version of the original signal, with an effective sampling rate *F*_eff_ ≤ *F*_S_. Of course, the samples in the subsampled signal would occur stochastically rather than regularly in time. Therefore, the effects of the present stochastic sampling mechanism on information transmission may be more easily understood by analyzing its effects on the effective sampling rate. To do it, let us assume for convenience a constant probability, *p*[*i*], along the signal duration. For an original signal with *N*_S_ samples, the *average* number of samples in the reconstructed signal, would be *p* × *N*_S_. In other words, for an original signal sampled at a rate *F*_S_, the *average* effective sampling rate, *F*_eff_, would be:

(A7) $$F_{eff}=p\times F_{S}$$

Figure A1 shows that a large *N* would be required for *p* = 1, hence for *F*_eff_ = *F*_S_, over a wide range of sample amplitudes. This case is illustrated by the horizontal line ➀ in Figure A1. In this case, *N* = 10,000 stochastic samplers would be required for *p* = 1 (hence for *F*_eff_ = *F*_S_) over a ~60-dB sample amplitude range, from just below 10^−3^ to 1. Figure A1 further shows that reducing *N* reduces the amplitude range over which *p* = 1 or *F*_eff_ = *F*_S_. In other words, it reduces the amplitude range over which the frequency content in the subsampled reconstructed signal approximates the original one.

Another way to understand the effects of reducing *N* would be to consider two sinusoidal stimuli of different frequencies with identical amplitudes of, say, 0.01. This case is illustrated by the vertical line ➁ in Figure A1. According to Equation A7, in this case, *F*_eff_ would be equal to *F*_S_ and ~0.1*F*_S_ for *N* = 1000 and *N* = 10, respectively. Therefore, according to Nyquist-Shannon's sampling theorem, the highest frequency that would be correctly represented in the reconstructed signal would be ten times lower for *N* = 10 than for *N* = 1000.

In summary, *N* determines the effective average sampling rate of the present stochastic sampling algorithm as well as the range of sample amplitudes over which such effective sampling rate applies. For a stimulus with a given sample-amplitude range and frequency bandwidth, reducing *N simultaneously* degrades the quality of the representation of low-amplitude and high-frequency components of the stimulus. Or, conversely, it reduces the dynamic range over which high-frequency content can be accurately represented.
